# Facteurs associés aux décès maternels au Centre hospitalier universitaire de Tengandogo, Ouagadougou, Burkina Faso, 2017-2022

**DOI:** 10.48327/mtsi.v6i2.2026.722

**Published:** 2026-06-30

**Authors:** Wedminère Noélie ZOUNGRANA-YAMEOGO, Vincent KABORE, Serge Alain TOUGMA, Abdoulaye Hama DIALLO, Aristide DJIGUIMDE, Dieudonné HIEN, Anthony SOME, Abdoulaye SO, Fidèle KAFANDO, Aida COMBARY, Mandata YANKINE, Dominique Hélène Laurel YABRE, Ali OUEDRAOGO

**Affiliations:** 1 Département de santé publique, Centre hospitalier universitaire (CHU) de Tengandogo, Ouagadougou, Burkina Faso; 2 Université Joseph Ki-Zerbo, Ouagadougou, Burkina Faso; 3 Centre des opérations de réponses aux urgences sanitaires, ministère de la Santé, Ouagadougou, Burkina Faso; 4 Département de gynécologie obstétrique, CHU de Tengandogo, Ouagadougou, Burkina Faso; 5 Institut de recherche en Sciences de la santé, Ouagadougou, Burkina Faso; 6 Direction de la qualité, CHU de Tengandogo, Ouagadougou, Burkina Faso; 7 Département des laboratoires et de la pharmacie, Ouagadougou, Burkina Faso

**Keywords:** Facteurs associés, Décès maternels, Hôpital, Burkina Faso, Afrique subsaharienne, Associated factors, Maternal deaths, Hospital, Burkina Faso, Sub-Saharan Africa

## Abstract

**Introduction:**

Avec un ratio de mortalité maternelle de 198 pour 100 000 naissances vivantes, le Burkina Faso figure parmi les pays africains ayant un taux élevé de mortalité maternelle et où la santé de la mère demeure une préoccupation majeure. Plusieurs facteurs sous-tendent cette mortalité. L’objectif de notre étude était d’identifier les facteurs associés aux décès maternels dans un hôpital de référence du Burkina Faso, le Centre hospitalier universitaire (CHU) de Tengandogo.

**Méthodes:**

Il s’est agi d’une étude rétrospective de cas témoins. Les dossiers sélectionnés concernaient les femmes admises au service de maternité et de réanimation entre le 1er janvier 2017 et le 31 décembre 2022. Les cas représentaient toutes les femmes admises pour une complication en rapport avec la grossesse et qui sont décédées. Les témoins étaient choisis de manière aléatoire parmi les femmes reçues pendant la période de l’étude pour accouchement et/ou complications liées à la grossesse, et qui n'étaient pas décédées au cours de la grossesse ou dans les 42 jours suivant son issue. Notre échantillon était constitué d’un cas pour deux témoins. Une régression logistique nous a permis d’identifier les facteurs associés.

**Résultats:**

Au total, 146 décès maternels ont été enregistrés mais 124 cas (22 dossiers inexploitables) et 248 témoins ont été analysés. Le ratio de mortalité maternelle intrahospitalière était de 1 960 pour 100 000 naissances vivantes (146/7 450). Chez les femmes décédées, l’âge moyen était de 28,5 ± 7,1 ans. Parmi elles, 73 (58,9 %) résidaient en milieu rural, 109 (87,9 %) étaient non salariées, 118 (84,7 %) avaient été référées par une formation sanitaire de niveau primaire ou secondaire, 106 (89,1 %) avaient été transportées par ambulance, 67 (54 %) étaient multipares, et 68 (57,1 %) avaient accouché par voie basse. La prééclampsie/ éclampsie était marquée chez 15 % des femmes et l’hématome rétro-placentaire (HRP) chez 13 % d’entre elles. Les facteurs indépendamment associés aux décès maternels étaient l’HRP (OR = 4,6 IC 95 % [1,6-12,7]), le moyen de transport par ambulance (OR = 3,8; IC 95 % [1,9-7,9]), et l’accouchement par voie basse (OR = 2,6; IC 95 % [1,5-4,5]).

**Conclusion:**

Le ratio de mortalité maternelle intrahospitalière est élevé. Un renforcement des compétences des gynécologues sur la détection précoce de l’HRP et la gestion des hémorragies est nécessaire. Un renforcement de la capacité de détection des complications pendant le suivi de la grossesse et pendant le travail de l’accouchement pour une meilleure décision obstétricale est également indispensable. Le ministère de la Santé devrait veiller aux bonnes conditions d’évacuation des patientes.

## Abstract (en)

**Introduction:**

Burkina Faso ranks among the African countries with high maternal mortality rates, with a ratio of 198 per 100,000 live births. Maternal health remains a major concern in this country. Several factors contribute to this mortality rate. Our study aimed to identify factors associated with maternal deaths at Tengandogo University Hospital, a referral hospital in Burkina Faso.

**Methods:**

This was a retrospective case-control study. We selected records pertaining to women admitted to the maternity and intensive care units between January 1, 2017, and December 31, 2022. The cases included all women admitted for a pregnancy-related complication who subsequently died. The control group was randomly selected from women admitted for childbirth and/ or pregnancy-related complications during the study period who did not die during pregnancy or within 42 days after delivery. Our sample consisted of one case for every two controls. Logistic regression was used to identify associated factors.

**Results:**

A total of 146 deaths were recorded, but 124 cases (22 records were unusable) and 248 controls were analyzed. The indoor maternal mortality ratio was 1,960 per 100,000 live births (146/7,450). The mean age of the women who died was 28.5 ± 7.1 years. Of those women, 73 (58.9%) lived in rural areas, 109 (87.9%) were unemployed, 118 (84.7%) were referred from a primary or secondary health facility, 106 (89.1%) were transported by ambulance, 67 (54%) were multiparous, and 68 (57.1%) delivered vaginally. Preeclampsia/eclampsia was present in 15% of the women and retroplacental hematoma (RPH) was present in 13%. The factors independently associated with maternal death were RPH (OR = 4.6, 95% CI [1.6–12.7]), ambulance transport (OR = 3.8, 95% CI [1.9–7.9]), and vaginal delivery (OR = 2.6, 95% CI [1.5–4.5]).

**Conclusion:**

The indoor maternal mortality rate is high. It is necessary to strengthen obstetricians’ skills in the early detection of RPH and hemorrhage management. Improving the ability to detect complications during prenatal care and labor and delivery is also essential for better obstetric decision-making. The Ministry of Health should ensure adequate conditions for patient evacuation.

**Figure:**
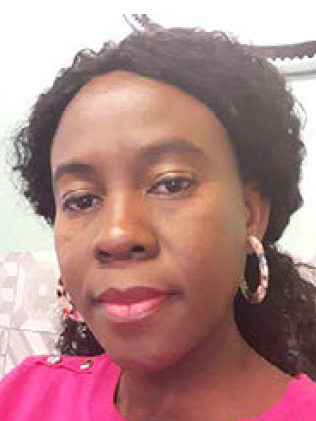

## Introduction

La mortalité maternelle est définie comme le décès d’une femme au cours de la grossesse ou dans un délai de 42 jours après son achèvement pour une cause quelconque déterminée ou aggravée par la grossesse ou les soins qu’elle a reçus, mais ni fortuite ni accidentelle [4]. La mortalité maternelle est reconnue comme l’un des meilleurs indicateurs du niveau de développement des systèmes de santé et des inégalités sociales en matière d’accès aux soins. Cet indicateur est intégré dans l’indice de développement humain publié chaque année par le Programme des Nations Unies pour le développement [15]. En 2023, 260 000 femmes sont décédées dans le monde pendant ou après une grossesse ou un accouchement [9]. Environ 90 % de ces décès sont survenus dans les pays à revenu faible ou intermédiaire [9].

Le Burkina Faso est un pays sahélien enclavé situé en Afrique de l’Ouest, dont 41,4 % de la population vit en dessous du seuil de pauvreté [5]. Son indice de développement humain en 2022 est de 0,449. Il est classé 184^e^ sur 189 pays avec un PIB/habitant de l’ordre de 809 € [13]. Le dernier recensement de la population et de l’habitation du Burkina Faso a dénombré 20 505 155 habitants en 2019 [6]. Le taux de natalité est de 31,64 pour 1 000 en 2023 et le taux de fécondité de 4,19 enfants/femme [16]. Le Burkina Faso figure parmi les pays africains ayant un ratio élevé de mortalité maternelle (198 décès pour 100 000 naissances vivantes [5]), et où la santé de la mère demeure une préoccupation majeure. Plusieurs facteurs associés aux décès maternels ont déjà été décrits par des auteurs dans des pays à revenu faible ou intermédiaire, notamment : les facteurs environnementaux; le milieu de résidence; les facteurs institutionnels; le délai (de 5 jours et plus) entre l’apparition des symptômes et l’admission à l’hôpital; le transport non médicalisé; et le « troisième retard » (retard de prise en charge de la parturiente [11]). D’autres auteurs ont décrit des causes directes de ces décès, telles que les hémorragies [1]. L’éclampsie, les infections, les complications liées aux avortements clandestins ont été également décrites comme facteurs associés aux décès maternels [3,9].

Plusieurs interventions ont été mises en place pour réduire le taux de mortalité maternelle dans le monde et en Afrique subsaharienne [12,14]. Malgré ces efforts, le ratio de mortalité maternelle reste élevé.

Le Centre hospitalier universitaire de Tengandogo (CHUT) possède un service d’obstétrique et de réanimation offrant des soins de recours pour des cas évacués depuis les centres de 1^er^ et 2^e^ niveaux de la pyramide sanitaire.

Entre 2017 et 2022, selon ses rapports annuels d’activités, le CHUT a enregistré 7 450 naissances vivantes et 146 décès maternels. Les ratios de mortalité maternelle intrahospitalière ont varié selon les années entre 407 et 2 851 décès pour 100 000 naissances vivantes. Cette variation s’explique en partie par celle de la fréquentation hospitalière, consécutive à la mise en œuvre, en novembre 2017, de la politique de gratuité des soins pour les femmes enceintes et les enfants de moins de 5 ans. Cependant, la mortalité intrahospitalière est largement au-dessus du ratio national.

Á ce jour, aucune étude sur les facteurs associés à ces décès maternels n’a été réalisée au CHUT. L’objet de la présente étude est de combler cette lacune, et de contribuer à réduire l’incidence de ces décès.

## Méthodes

Nous avons mené une étude cas témoins dans les services de gynécologie-obstétrique et de réanimation polyvalente du CHUT de la ville de Ouagadougou, l’un des trois centres de références de niveau 3, sommet de la pyramide sanitaire du Burkina Faso.

Cet hôpital a officiellement ouvert ses portes et débuté la prise en charge des urgences gynécologiques et obstétricales et des hospitalisations le 8 octobre 2012. Sa capacité est de 600 lits, mais seuls 338 étaient en service en 2022. Le nombre de consultations s’élevait à 38 444 en 2022, le nombre d’admissions à 11 463. Le service de gynécologie-obstétrique comporte les urgences gynécologiques et obstétricales, un bloc opératoire, la consultation externe et une unité d’hospitalisation. C’est un service spécialisé qui reçoit des patientes référées par les structures sanitaires publiques et privées de la ville de Ouagadougou, de la province du Kadiogo et des provinces environnantes. Le personnel est constitué de 8 médecins gynécologues obstétriciens et de 33 sages-femmes et maïeuticiens d’État. Le service de réanimation polyvalente a une capacité totale de 24 lits, dont 12 opérationnels. L’équipe médicale comprenait au moment de l’étude, cinq médecins anesthésistes-réanimateurs, six médecins en spécialisation en anesthésie-réanimation et deux médecins généralistes.

Les données utilisées pour cette étude ont concerné la période allant du 1^er^ janvier 2017 au 31 décembre 2022. La population retenue était les femmes admises dans ces deux services pour complication en rapport avec la grossesse, l’accouchement et le *post-partum*.

Les cas étaient constitués des patientes décédées au cours de la grossesse ou dans un délai de 42 jours après sa fin dans le service de gynécologie obstétrique ou dans le service de réanimation polyvalente, pendant la période de l’étude. Les témoins ont été choisis de façon aléatoire parmi les femmes reçues pour accouchement et/ou complications liées à la grossesse et qui sont ressorties vivantes. Chacune de ces femmes était enregistrée dans un registre d’admission avec un numéro de dossier unique que nous avons transcrit dans Excel. Avec la fonction ALEA, nous avons procédé au tirage au sort des témoins nécessaires pour notre analyse.

Un échantillonnage exhaustif pour les cas a été effectué, et deux témoins par cas ont été sélectionnés. Les données ont été collectées par un étudiant de 8^e^ année de médecine dans le cadre de sa thèse d’exercice.

La variable dépendante était le « décès maternel », codée en oui ou non. Les variables indépendantes étaient : l’âge, la résidence, la profession, le statut matrimonial, le niveau d’instruction, le mode d’admission directe (la patiente s’est rendue ellemême à l’hôpital accompagnée ou non par un membre de la famille), le mode d’admission par référence (la patiente est passée d’abord par une formation sanitaire de niveau primaire ou secondaire, puis adressée dans un centre hospitalier pour une meilleure prise en charge), la gestité, la parité, les antécédents d’avortement ou de césarienne, le moyen de transport utilisé pour se rendre à l’hôpital, le nombre de consultations prénatales, le mode d’accouchement, la prééclampsie/éclampsie, l’hématome rétro-placentaire (HRP), la présence d’un *placenta prævia*, le paludisme grave. Par paludisme grave, était entendu un cas de paludisme à *Plasmodium falciparum* avec au moins un des signes de gravité suivants : troubles de la conscience ou léthargie, convulsions répétées (plus de 2 épisodes/24h), pâleur sévère, prostration, détresse respiratoire, œdème aigu du poumon, choc ou collapsus cardio-vasculaire, hémoglobinurie, ictère franc, hémorragies spontanées, oligo-anurie.

Les informations ont été recueillies à partir des dossiers cliniques et des registres d’admission. Les données ont été enregistrées sur l’application Kobocollect sur téléphone mobile, dans sa version 2022.2.3. L’analyse a été réalisée avec le logiciel STATA dans sa version 15.

Nous avons utilisé la moyenne pour décrire les variables quantitatives et la proportion pour les variables qualitatives, et avons procédé à l’analyse en deux étapes, en utilisant la régression logistique : la 1^re^ était l’analyse univariée. Les variables associées au décès avec un niveau de signification inférieur à 20 % ont été introduites dans le modèle multivarié; la 2^e^ étape était l’analyse multivariée pour laquelle nous avons utilisé la méthode pas à pas descendante. Les variables ont été exclues du modèle au fur et à mesure que leur liaison au décès était à un niveau de signification supérieur à 5 %. Nous avons gardé dans le modèle final toutes les variables associées au décès avec un niveau de signification inférieur à 5 %.

L’autorisation du directeur général du CHUT pour la conduite de cette étude a été obtenue. Toutes les dispositions ont été prises pour garantir l’anonymat et la confidentialité des patientes.

## Résultats

Au cours de la période de l’étude (2017-2022), 7 696 patientes ont été admises pour complications obstétricales au service de gynécologie-obstétrique du CHUT. Durant la même période, 7 450 naissances vivantes y ont été enregistrées, ainsi que 146 décès maternels à la maternité ou au service de réanimation polyvalente.

Le ratio de mortalité maternelle intrahospitalière était donc de 1 960 pour 100 000 naissances vivantes (146/7 450). Sur les 146 décès, 124 dossiers ont pu être exploités, 22 étant inexploitables car incomplets (5 dossiers en lien avec des femmes arrivées décédées, 13 patientes enregistrées dont les dossiers n’ont pas été retrouvés, 4 dossiers pour lesquels plus de 50 % des informations étaient manquantes).

Chez les cas, 106 patientes (89,1 %) ont utilisé l’ambulance pour se rendre à l’hôpital et 13 autres (10,9 %) un moyen de transport personnel. Pour les témoins, 136 patientes (69 %) ont utilisé l’ambulance et 61 patientes (31 %) un moyen de transport personnel. Chez les cas, 6 (4,8 %) patientes se sont rendues directement elles-mêmes à l’hôpital, tandis que 118 (95,2 %) sont passées d’abord dans une formation sanitaire de niveau primaire ou secondaire qui les a référées au CHUT. Chez les témoins, 51 (20,6 %) patientes se sont rendues directement à l’hôpital, et 197 (79,4 %) ont été référées à partir d’une formation sanitaire périphérique.

Les autres caractéristiques des patientes (socio-démographiques et médico-gynécologiques) sont présentées dans les Tableaux II et II.

**Tableau I:** Caractéristiques sociodémographiques des femmes incluses dans l’étude au Centre hospitalier universitaire de Tengandogo, 2017-2022

Caractéristiques	Cas n (%)	Témoins n (%)	p
Âge			
15-25	46 (37,1)	102 (41,1)	0,7367
26-35	54 (43,6)	99 (39,9)	
≥ 36	24 (19,4)	47 (19)	
Résidence			
rurale	73 (58,9)	94 (37,9)	0,000
urbaine	51 (41,1)	154 (62,1)	
Profession			
non salariée	109 (87,9)	188 (84,7)	0,0174
salariée	8 (4,8)	34 (15,3)	
Situation matrimoniale			
en couple	110 (88,7)	220 (88,7)	1,000
célibataire	14 (11,3)	28 (11,3)	
Niveau d'instruction			
non scolarisée	55 (44,4)	72 (29)	0,0055
primaire- secondaire	65 (52,4)	156 (62,9)	
supérieur	4 (3,2)	20 (8,1)	

**Tableau II:** Caractéristiques médico-obstétricales des patientes incluses dans l’étude au Centre hospitalier universitaire de Tengandogo, 2017-2022

Caractéristiques	Cas n (%)	Témoins n (%)	p
Gestité			
primigeste (1)	32 (25,8)	85 (34,3)	0,0104
paucigeste (2-3)	33 (26,6)	85 (34,3)	
multigeste (≥4)	59 (47,6)	78 (31,5)	
Parité			
primipares (1)	22 (17,7)	47 (19)	0,7976
paucipares (2-3)	35 (28,2)	62 (25)	
multipares (≥4)	67 (54)	139 (56,1)	
Antécédent d'avortement			
oui	17 (13,7)	40 (16,1)	0,5383
non	107 (83,9)	208 (83,9)	
Antécédent de césarienne			
oui	14 (11,3)	64 (25,8)	0,0007
non	110 (88,7)	184 (74,2)	
Nombre de consultations prénatales (CPN)			
1	11 (10,6)	1 (0,4)	0,000
2-3	37 (35,6)	78 (32,4)	
≥4	56 (53,9)	162 (67,2)	
Mode d'accouchement			
voie basse	68 (57,1)	90 (36,7)	0,0002
césarienne	51 (42,9)	155 (63,3)	
Prééclampsie-éclampsie			
oui	19 (15,3)	31 (12,5)	0,8243
non	105 (84,7)	217 (87,5)	
Hématome rétro-placentaire			
oui	16 (12,9)	7 (2,8)	0,000
non	108 (87,1)	241 (97,2)	
Grossesse extra-utérine			
oui	1 (0,8)	1 (0,4)	0,6264
non	123 (99,2)	247 (99,6)	
Placenta prævia			
oui	2 (1,6)	3 (1,2)	0,7537
non	122 (98,3)	245 (98,8)	
Paludisme grave			
oui	6 (4)	2 (0,8)	0,0388
non	118 (95,6)	246 (99,2)	

Les variables associées au décès avec un niveau de signification inférieur à 0,2 ont été prises en compte pour l’analyse multivariée. Il s’agissait de la résidence, la profession, le niveau d’instruction, le moyen de transport utilisé par la patiente, le mode d’admission, le paludisme grave, la gestité, la parité, le nombre de consultations prénatales (CPN), le mode d’accouchement et le HRP. Les détails sur l’analyse univariée sont présentés dans les Tableaux III et IV.

**Tableau III:** Analyse univariée des facteurs potentiels (caractéristiques sociodémographiques) associés au décès maternel au Centre hospitalier universitaire de Tengandogo, 2017-2022

Variables	Cas n (%)	Témoins n (%)	ORb [IC95%]	p
Âge			
15-25	46 (37,1)	102 (41,1)	1	0,7367
26-35	54 (43,6)	99 (39,9)	1,2 [0,7 -2]	
≥ 36	24 (19,4)	47 (19)	1,1 [0,6-2,1]	
Résidence			
rurale	73 (58,9)	94 (37,9)	1	0,0001
urbaine	51 (41,1)	154 (62,1)	0,42 [0,27-0,66]	
Profession			
non salariée	109 (93,2)	188 (84,7)	1	0,07
salariée	8 (6,8)	34 (15,3)	0,46 [0,19-1,10]	
Situation matrimoniale			
en couple	110 (88,7)	220 (88,7)	1	1
célibataire	14 (11,3)	28 (11,3)	1 [0,6-2]	
Niveau d'instruction			
non scolarisée	55 (44,4)	72 (29)	1	0,0055
primaire- secondaire	65 (52,4)	156 (62,9)	0,54 [0,34-0,86]	
supérieur	4 (3,2)	20 (8,1)	0,26 [0,08-0,81]	
Mode d'admission			
s'est rendue elle-même au CHUT	6 (4,8)	51 (20,6)	1	0,0001
référée par une formation sanitaire	118 (95,2)	197 (79,4)	5,1 [2,1-12,2]	
Moyen de transport			
ambulance	106 (89,1)	136 (70)	1	
moyen de transport personnel	133 (10,9)	61 (31)	0,3 [0,1-0,5]	

ORb = Odds Ratio brut

**Tableau IV:** Analyse univariée des facteurs potentiels (médico-gynécologiques) associés au décès maternel au Centre hospitalier universitaire de Tengandogo, 2017-2022

Variables	Cas n (%)	Témoins n (%)	ORb [IC95%]	p
Gestité				
primigeste (1)	32 (25,8)	85 (34,3)	1	0,0104
paucigeste (2-3)	33 (26,6)	85 (34,3)	1,03 [0,6-1,8]	
multigeste (≥4)	59 (47,6)	78 (31,5)	2 [1,2-3,4]	
Parité				
primipares (1)	22 (17,7)	47 (19)	0,83 [0,42-1,6]	0,7976
paucipares (2-3)	35 (28,2)	62 (25)	1	
multipares (≥4)	67 (54)	139 (56)	0,85 [0,5-1,41]	
Antécédent d'avortement				
oui	17 (13,7)	40 (16,1)	1	0,5383
non	107 (83,9)	208 (83,9)	0,8 [0,44-1,53]	
Antécédent de césarienne				
oui	14 (11,3)	64 (25,8)	0,37 [0,19-0,68]	0,0007
non	110 (88,7)	184 (74,2)	1	
Nombre de consultations prénatales (CPN)				
1	11 (10,6)	1 (0,4)	1	0
2-3	37 (35,6)	78 (32,3)	0,04 [0,005-0,4]	
≥4	56 (53,9)	162 (67,2)	0,03 [0,003-0,2]	
Mode d'accouchement				
voie basse	68 (57,1)	90 (36,7)	2,4 [1,5-3,7]	0,0002
césarienne	51 (42,9)	155 (63,3)	1	
Prééclampsie-éclampsie				
oui	19 (15,3)	31 (12,5)	1,3 [0,7-2,3]	0,8243
non	105 (84,7)	217 (87,5)	1	
Hématome rétro-placentaire				
oui	16 (12,9)	7 (2,8)	5,1 [2-12,8]	0
non	108 (87,1)	241 (97,2)	1	
Grossesse extra-utérine				
oui	1 (0,8)	1 (0,4)	2 [0,1-32,4]	0,6264
non	123 (99,2)	247 (99,6)	1	
Placenta prævia				
oui	2 (1,6)	3 (1,2)	1,3 [0,2-8,1]	0,7537
non	122 (98,4)	245 (98,8)	1	
Paludisme grave				
oui	6 (4,8)	2 (1,2)	4,2 [1-16,9]	0,0388
non	118 (95,2)	245 (98,8)	1	

ORb = Odds Ratio brut

En analyse multivariée, les variables indépendamment associées au décès maternel étaient le HRP (OR = 4,6; IC 95 % [1,6-12,7]), le moyen de transport par ambulance (OR = 3,8; IC 95 % [1,9-7,9]) et l’accouchement par voie basse (OR = 2,6; IC 95 % [1,5-4,5]) (Tableau V).

**Tableau V:** Analyse multivariée des facteurs associés au décès maternel au Centre hospitalier universitaire de Tengandogo 2017-2022

Variables	Cas n (%)	Témoins n (%)	ORb [IC95%]	p	ORa [IC95%]	p
Moyen de transport			
ambulance	106 (89,1)	136 (69)	3,7[1,9-7]	0	3,8 [1,9-7,9]	0, 000
moyen de transport personnel	13 (10,9)	61 (31)	1		1	
Mode d'accouchement			
voie basse	68 (57,1)	90 (36,7)	2,4[1,5-3,7]	0,0002	2,6 [1,5-4,5]	0
césarienne	51 (42,9)	155 (63,3)	1		1	
Hématome rétro-placentaire			
oui	16 (12,9)	7 (2,8)	5,1 [2-12,8]	0	4,6 [1,6-12,7]	0,004
non	108 (87,1)	241 (97,2)	1		1	

ORb = Odds Ratio brut

ORa = Odds Ratio ajusté

## Discussion

L’objectif principal de cette étude était d’identifier les facteurs associés aux décès maternels au CHUT, établissement de référence de 3^e^ niveau dans la pyramide sanitaire nationale. Rappelons que ce centre accueille des patientes évacuées ou référées depuis les structures sanitaires de 1^er^ et 2^e^ niveaux, notamment les centres de santé et de promotion sociale, les centres médicaux avec antenne chirurgicale, les hôpitaux régionaux et les cliniques privées, pour une prise en charge spécialisée des complications obstétricales graves. La collecte des données, réalisée dans le cadre d’une étude rétrospective, a été confrontée à plusieurs limites méthodologiques. Nous avons cherché à minimiser les biais de recueil de données en utilisant des variables catégoriques simples à identifier et en les codant (0 ou 1) chaque fois que possible. Nous avons exclu les dossiers cliniques incomplets ou introuvables. Par ailleurs, pour certaines variables (moyen de transport, profession), il existait des données manquantes. En outre, le caractère monocentrique de l’étude a pu restreindre la généralisation des résultats, bien que le CHUT reçoive des patientes provenant de l’ensemble du territoire national. Les pratiques cliniques, les ressources disponibles et les conditions d’hospitalisation pouvant varier d’un établissement à l’autre, ces éléments doivent être pris en compte dans l’interprétation des résultats. Malgré ces limites, nous avons pu calculer le ratio de mortalité maternelle intrahospitalière à partir des décès maternels enregistrés de 2017 à 2022 au CHUT et identifier les facteurs associés aux décès maternels. Le ratio observé est élevé dans ce centre où sont référées les complications obstétricales les plus graves. Cette situation pourrait aussi être due à l’insuffisance du plateau technique de l’établissement (un seul bloc opératoire fonctionnel, ce qui contribue à allonger le délai de prise en charge de certaines urgences). Le manque de personnel oblige à mutualiser l’équipe de garde avec le personnel de la chirurgie. À cela s’ajoutent d’une part le retard de consultation – ou de référencement – des patientes ayant une complication et, d’autre part, le nombre insuffisant de consultations prénatales [17].

Le HRP a été également décrit comme facteur de risque par Bouvier *et al*. et Say *et al*. [2,12]. Le risque d’hémorragie abondante nécessite une prise en charge urgente, une disponibilité des produits sanguins labiles et, souvent, des mesures de réanimation urgentes. Cela n’est généralement pas le cas au Burkina Faso où le personnel qualifié et le matériel de réanimation sont insuffisants. L’étude n’ayant pas intégré l’analyse des délais (variable non prise en compte) de prise en charge des HRP, il est probable que ces urgences aient été traitées hors délais optimaux, comme c’est fréquemment observé dans notre contexte. Ce retard dans la réponse médicale, souvent lié à des contraintes organisationnelles et logistiques, pourrait contribuer à expliquer que le HRP apparaisse comme un facteur significativement associé à la mortalité maternelle.

Notre travail a retrouvé une association entre le mode d’accouchement par voie basse et le décès maternel. Ce mode d’accouchement ne constitue pas un risque en soi, la majorité des accouchements par voie basse se déroulant bien. Selon le rapport annuel d’activités du CHUT de 2022, sur 2 758 accouchements, 1 195 étaient par voie basse (43,32 %) et 1 563 par césarienne, avec 59 décès maternels toutes voies confondues, soit 2 % de décès pour ces deux modes d’accouchement. Cependant, une issue défavorable pour la mère et pour l’enfant peut se produire lorsque certaines complications obstétricales ne sont pas détectées à temps pendant le suivi de la grossesse et au moment de l’accouchement, et qu’une bonne décision obstétricale n’est pas prise.

À cela s’ajoute la disponibilité ou non des produits nécessaires pour la gestion de certaines urgences. Dans notre étude, 15 % des patientes décédées avaient été admises pour prééclampsie/éclampsie. Des audits de décès maternels réalisés au CHUT avaient souligné la non-disponibilité de l’alpha méthyl dopa, obligeant les accompagnants à parcourir des kilomètres pour en acheter, l’hôpital n’étant pas situé en ville. Cela a pu augmenter le délai de prise en charge. Par ailleurs, 95,2 % des patientes sont passées d’abord dans une formation sanitaire de niveau inférieur avant d’être référées pour insuffisance de plateau technique (absence de bloc opératoire, bloc opératoire en panne) et/ ou de ressources humaines qualifiées (absence ou insuffisance de gynécologues ou de médecins réanimateurs, etc.). Notre étude n’a malheureusement pas pris en compte le délai entre l’admission de ces patientes dans ces formations sanitaires et la décision de référence, alors que ce délai peut influer sur le risque de survenue de décès.

Il faut aussi mentionner le temps d’attente des ambulances, souvent en nombre insuffisant. Le transport par ambulance comme facteur associé au décès s’explique par un biais : les ambulances transportent les cas les plus graves. Plusieurs audits réalisés ont montré qu’elles sont en quantité insuffisante, très souvent en panne dans la plupart des formations sanitaires et, en majorité, non médicalisées. Il arrive aussi que plusieurs femmes soient transportées dans la même ambulance. Le renforcement du plateau technique dans les formations sanitaires de 1^er^ et 2^e^ niveaux, et l’augmentation du nombre d’ambulances médicalisées sont des leviers essentiels pour améliorer la prise en charge des urgences obstétricales et réduire l’incidence des décès maternels.

Nos résultats diffèrent de ceux de Raoul *et al*. qui avaient trouvé que les facteurs associés aux décès maternels étaient le lieu de résidence, le délai (de cinq jours et plus) entre l’apparition des symptômes et l’admission à l’hôpital, le transport non médicalisé et « le troisième retard » [10]. Mahbouli *et al*. ont mis en évidence que la primiparité, la multiparité, les conditions socioéconomiques défavorables et le nombre insuffisant de consultations prénatales constituaient les principaux facteurs associés à la mortalité maternelle [8]. D’autres facteurs tels que l’éclampsie, l’absence de consultations prénatales, la présence de comorbidités, ont été décrits par Yego *et al*. dans une étude analytique réalisée au Kenya et portant sur 450 femmes [16].

Pour réduire la mortalité maternelle au Burkina Faso, plusieurs stratégies peuvent être adoptées : la participation des agents de santé à base communautaire à la reconnaissance des signes de danger; la médicalisation des références obstétricales; la réduction du « troisième retard » (décision de prise en charge) dans les structures de références. Ceci passe par l’amélioration des conditions de pratique de la césarienne dans des délais raisonnables en cas d’accouchement à risque. Le bénéfice de la césarienne a déjà été présenté par plusieurs acteurs [7,13,17].

## Conclusion

Le ratio de mortalité maternelle intrahospitalière est élevé au CHUT, centre de référence de 3^e^ niveau. Le HRP, les mauvaises conditions d’évacuation par ambulance et les complications liées à l’accouchement par voie basse sont les facteurs indépendamment associés aux décès maternels. Le CHUT doit renforcer le service de gynécologie obstétrique en ressources humaines, tant en quantité qu’en qualité, afin de permettre un fonctionnement autonome.

Par ailleurs, la disponibilité des produits d’urgence est indispensable. L’ouverture d’un second bloc opératoire réduirait les délais d’intervention obstétricale des patientes. Un renforcement continu des prestataires des différents centres de santé de 1^er^ et 2^e^ niveaux (infirmiers, sage-femmes, gynécologues) en compétences et outils cliniques pour un diagnostic précoce et une bonne prise en charge des complications obstétricales est nécessaire pour réduire les évacuations des patientes qui se font généralement dans des conditions inappropriées. Il en est de même de la gestion de l’hémorragie en *per* ou *postpartum* qui demande d’améliorer l’offre en produits sanguins labiles. Enfin, l’augmentation du nombre d’ambulances correctement médicalisées est fortement recommandée.

## Remerciements

Nous remercions le Directeur général du Centre hospitalier universitaire de Tengandogo d’avoir accordé l’autorisation de collecter des données.

## Financement

Cette étude n’a reçu aucun financement.

## Contribution des auteurs et autrices

Le Dr Vincent Kaboré a collecté les données dans le cadre de sa thèse d’exercice qui a été dirigée par le Dr Wedminère Noélie Zoungrana-Yameogo et le Pr Ali Ouedraogo. Les analyses et la rédaction de la version initiale ont été effectuées par la Dr Wedminère Noélie Zoungrana-Yameogo. La première version a été partagée avec Serge Alain Tougma, Abdoulaye Hama Diallo, Aristide Djiguimde, Dieudonné Hien, Anthony Some, Abdoulaye So, Fidèle Kafando, Mandeta Yankine, Aida Combary, Dominique Hélène Laurel Yabre et le Pr Ouedraogo Ali pour lecture.

## Déclaration de liens d’intérêts

Aucun lien d’intérêt n’a été déclaré.
