# Supplementary material for: Factors associated with maternal deaths at Tengandogo University Hospital, Ouagadougou, Burkina Faso, 2017–2022
Source: Med Trop Sante Int. 2026 Jun 30;6(2):mtsi.v6i2.2026.722. [Article in French] doi: 10.48327/mtsi.v6i2.2026.722 (PMC13423298; doi:10.48327/mtsi.v6i2.2026.722)
Supplement: Related texts

# Research article: Factors associated with maternal deaths at Tengandogo University Hospital, Ouagadougou, Burkina Faso, 2017–2022

## Introduction

Maternal mortality is defined as the death of a woman during pregnancy or within 42 days after its completion due to any cause that is related to the pregnancy or the care she received. This cause must not be fortuitous or accidental [4]. It is recognized as one of the best indicators of the level of development of health systems and of social inequalities in access to care. It is included in the Human Development Index, which is published annually by the United Nations Development Programme [15]. In 2023, an estimated 260,000 women worldwide died during or after pregnancy or childbirth [9]. Approximately 90% of these deaths occurred in low- and middle-income countries [9].

Burkina Faso is a landlocked Sahelian country in West Africa where 41.4% of the population lives below the poverty line [5]. In 2022, its Human Development Index was 0.449. Burkina Faso is ranked 184^th^ out of 189 countries, with a GDP per capita of approximately €809 [13]. The latest population census in Burkina Faso recorded 20,505,155 inhabitants in 2019 [6]. The birth rate was 31.64 per 1,000 people in 2023, and the total fertility rate was 4.19 children per woman [16]. Burkina Faso is among the African countries with a high maternal mortality ratio of 198 deaths per 100,000 live births [5], where maternal health remains a major concern. Several factors associated with maternal deaths have been described by researchers in low- and middle-income countries. These factors include environmental factors, place of residence, institutional factors, delays of five days or more between the onset of symptoms and hospital admission, nonmedical transport, and the “third delay” (delay in care for the parturient) [11]. Other authors have described direct causes of these deaths, such as hemorrhage [1]. Eclampsia, infections, and complications related to clandestine abortions have also been described as factors associated with maternal deaths [3,9]. Several interventions have been implemented worldwide and in sub-Saharan Africa to reduce the maternal mortality rate [12,14]. Despite these efforts, the maternal mortality ratio remains high. Tengandogo University Hospital (CHUT) has an obstetrics and intensive care unit that provides specialized care for cases referred from first- and second-level healthcare facilities.

According to its annual activity reports, between 2017 and 2022, the CHUT recorded 7,450 live births and 146 maternal deaths. Indoor maternal mortality rates varied by year, ranging from 407 to 2,851 deaths per 100,000 live births. This variation is partly explained by fluctuations in hospital admissions following the implementation of the free healthcare policy for pregnant women and children under five years of age in November 2017. However, the indoor mortality rate remains well above the national rate.

To date, no study has been conducted at the CHUT on the factors associated with these maternal deaths. The purpose of this study is to address this gap and help reduce the incidence of these deaths.

## Methods

We conducted a case-control study in the gynecology-obstetrics and general intensive care units of the CHUT in Ouagadougou, which is one of three Level 3 referral centers at the top of Burkina Faso’s healthcare pyramid.

The hospital officially opened on October 8, 2012, and began providing care for gynecological and obstetric emergencies and hospitalizations. Although it has a capacity of 600 beds, only 338 were in use in 2022. There were 38,444 outpatient visits and 11,463 admissions in 2022. The gynecology-obstetrics department includes a unit for gynecological and obstetric emergencies, an operating room, outpatient services, and an inpatient unit. This specialized department receives patients referred from public and private healthcare facilities in Ouagadougou, Kadiogo Province, and the surrounding area. The department’s staff consists of eight obstetrician-gynecologists and 33 state-certified midwives and obstetric nurses. The general intensive care unit has a total capacity of 24 beds, 12 of which are operational. At the time of the study, the medical team included five anesthesiologists and intensivists, six residents specializing in anesthesiology and intensive care, and two general practitioners.

The data used for this study covered the period from January 1, 2017, to December 31, 2022. The study population consisted of women admitted to these two units for complications related to pregnancy, childbirth, and the *postpartum* period.

Cases consisted of patients who died during pregnancy or within 42 days after its end in the obstetrics and gynecology department or general intensive care unit during the study period. Controls were randomly selected from women admitted for childbirth and/or pregnancy-related complications who were discharged alive. Each woman was recorded in an admission registry with a unique case number, which we entered into Excel. Using the RANDOM function, we randomly selected the necessary controls for our analysis.

Exhaustive sampling was performed for the cases, with two controls selected for each case. An eighth-year medical student collected the data as part of his thesis.

The dependent variable was “maternal death,” coded as “yes” or “no.” The independent variables were age; place of residence; occupation; marital status; educational level; mode of direct admission (whether the patient went to the hospital on her own or was accompanied by a family member); admission via referral (whether the patient first visited a primary or secondary health facility and was then referred to a hospital for better care); gestational age; parity; history of abortion or cesarean section; mode of transportation used to reach the hospital; number of prenatal visits; mode of delivery; preeclampsia/eclampsia; retroplacental hematoma (RPH); and presence of *placenta previa* or severe malaria. Severe malaria was defined as a case of *Plasmodium falciparum* malaria with at least one of the following signs of severity: impaired consciousness, lethargy, repeated seizures (more than two episodes in 24 hours), severe pallor, prostration, respiratory distress, acute pulmonary edema, shock, cardiovascular collapse, hemoglobinuria, overt jaundice, spontaneous hemorrhages, or oligoanuria.

Information was collected from clinical records and admission registries. Data were recorded using the Kobocollect mobile app, version 2022.2.3. The analysis was performed using Stata software, version 15.

We used the mean to describe quantitative variables and the proportion to describe qualitative variables. We conducted the analysis in two stages using logistic regression. First, we performed a univariate analysis. Variables associated with death at a significance level below 20% were included in the multivariate model. The second stage was the multivariate analysis, for which we used the stepwise downward method. Variables were excluded from the model when their association with death reached a significance level above 5%. In the final model, we retained all variables associated with death that were significant at a level below 5%.

We obtained authorization from the Director General of the CHUT to conduct this study. All measures were taken to ensure the anonymity and confidentiality of the patients.

## Results

During the study period (2017–2022), the CHUT’s Department of Obstetrics and Gynecology admitted 7,696 patients for obstetric complications. There were 7,450 live births and 146 maternal deaths in the maternity ward or general intensive care unit during the same period.

Therefore, the indoor maternal mortality rate was 1,960 per 100,000 live births (146/7,450). Of the 146 deaths, 124 records were analyzed. Twenty-two records were unusable due to being incomplete: five involving women pronounced dead on arrival, thirteen patients whose records could not be located, and four records missing more than 50% of information.

Among the analyzed cases, 106 patients (89.1%) arrived by ambulance and 13 (10.9%) arrived by personal vehicle. Among the control group, 136 patients (69%) used an ambulance, while 61 patients (31%) used personal transportation. Six (4.8%) cases went directly to the hospital, while 118 (95.2%) first visited a primary or secondary health facility that referred them to the CHUT. Among the controls, 51 patients (20.6%) went directly to the hospital, while 197 patients (79.4%) were referred from a peripheral health facility. The other characteristics of the patients (sociodemographic and medical-gynecological) are presented in Tables I and II.

**Table I:** Sociodemographic characteristics of the women included in the study at Tengandogo University Hospital, 2017–2022

Characteristics	Case n (%)	Control n (%)	p
Age			
15-25	46 (37,1)	102 (41,1)	0,7367
26-35	54 (43,6)	99 (39,9)	
≥ 36	24 (19,4)	47 (19)	
Residence			
rural	73 (58,9)	94 (37,9)	0,000
urban	51 (41,1)	154 (62,1)	
Occupation			
self-employed	109 (87,9)	188 (84,7)	0,0174
employed	8 (4,8)	34 (15,3)	
Marital status			
in a relationship	110 (88,7)	220 (88,7)	1,000
single	14 (11,3)	28 (11,3)	
Education level			
no formal education	55 (44,4)	72 (29)	0,0055
primary - secondary	65 (52,4)	156 (62,9)	
higher education	4 (3,2)	20 (8,1)	

**Table II:** Medical and obstetric characteristics of patients included in the study at Tengandogo University Hospital, 2017–2022

Characteristics	Case n (%)	Control n (%)	p
Gestational history			
primigravida (1)	32 (25,8)	85 (34,3)	0,0104
paucigravida (2-3)	33 (26,6)	85 (34,3)	
multigravida (≥4)	59 (47,6)	78 (31,5)	
Parity			
primiparous (1)	22 (17,7)	47 (19)	0,7976
pauciparous (2-3)	35 (28,2)	62 (25)	
multiparous (≥4)	67 (54)	139 (56,1)	
History of abortion			
yes	17 (13,7)	40 (16,1)	0,5383
no	107 (83,9)	208 (83,9)	
History of cesarean section			
yes	14 (11,3)	64 (25,8)	0,0007
no	110 (88,7)	184 (74,2)	
Number of prenatal visits (PNV)			
1	11 (10,6)	1 (0,4)	0,000
2-3	37 (35,6)	78 (32,4)	
≥4	56 (53,9)	162 (67,2)	
Mode of delivery			
vaginal delivery	68 (57,1)	90 (36,7)	0,0002
cesarean section	51 (42,9)	155 (63,3)	
Preeclampsia-eclampsia			
yes	19 (15,3)	31 (12,5)	0,8243
no	105 (84,7)	217 (87,5)	
Retroplacental hematoma			
yes	16 (12,9)	7 (2,8)	0,000
no	108 (87,1)	241 (97,2)	
Ectopic pregnancy			
yes	1 (0,8)	1 (0,4)	0,6264
no	123 (99,2)	247 (99,6)	
Placenta previa			
yes	2 (1,6)	3 (1,2)	0,7537
no	122 (98,3)	245 (98,8)	
Severe malaria			
yes	6 (4)	2 (0,8)	0,0388
no	118 (95,6)	246 (99,2)	

Variables associated with death and having a p-value less than 0.2 were included in the multivariate analysis. These variables included residence, occupation, educational level, mode of transportation, mode of admission, presence of severe malaria, gestational age, parity, number of antenatal care visits, mode of delivery, and RPH. Details on the univariate analysis are presented in Tables III and IV

**Table III:** Univariate analysis of potential factors (sociodemographic characteristics) associated with maternal mortality at Tengandogo University Hospital, 2017–2022

Variables	Case n (%)	Control n (%)	COR [95% CI]	p
Age				
15-25	46 (37,1)	102 (41,1)	1	0,7367
26-35	54 (43,6)	99 (39,9)	1,2 [0,7 -2]	
≥ 36	24 (19,4)	47 (19)	1,1 [0,6-2,1]	
Residence				
rural	73 (58,9)	94 (37,9)	1	0,0001
urban	51 (41,1)	154 (62,1)	0,42 [0,27-0,66]	
Occupation				
self-employed	109 (93,2)	188 (84,7)	1	0,07
employed	8 (6,8)	34 (15,3)	0,46 [0,19-1,10]	
Marital status				
in a relationship	110 (88,7)	220 (88,7)	1	1
single	14 (11,3)	28 (11,3)	1 [0,6-2]	
Education level				
no formal education	55 (44,4)	72 (29)	1	0,0055
primary - secondary	65 (52,4)	156 (62,9)	0,54 [0,34-0,86]	
higher education	4 (3,2)	20 (8,1)	0,26 [0,08-0,81]	
Admission mode				
self-presented to CHUT	6 (4,8)	51 (20,6)	1	0,0001
referred by a healthcare facility	118 (95,2)	197 (79,4)	5,1 [2,1-12,2]	
Mode of transport				
ambulance	106 (89,1)	136 (70)	1	
personal vehicle	133 (10,9)	61 (31)	0,3 [0,1-0,5]	

COR = Crude Odds Ratio

**Table IV:** Univariate analysis of potential factors (medical and gynecological) associated with maternal mortality at Tengandogo University Hospital, 2017–2022

Variables	Case n (%)	Control n (%)	COR [95%CI]	p
Gestational history				
primigravida (1)	32 (25,8)	85 (34,3)	1	0,0104
paucigravida (2-3)	33 (26,6)	85 (34,3)	1,03 [0,6-1,8]	
multigravida (≥4)	59 (47,6)	78 (31,5)	2 [1,2-3,4]	
Parity				
primiparous (1)	22 (17,7)	47 (19)	0,83 [0,42-1,6]	0,7976
pauciparous (2-3)	35 (28,2)	62 (25)	1	
multiparous (≥4)	67 (54)	139 (56)	0,85 [0,5-1,41]	
History of abortion				
yes	17 (13,7)	40 (16,1)	1	0,5383
no	107 (83,9)	208 (83,9)	0,8 [0,44-1,53]	
History of cesarean section				
yes	14 (11,3)	64 (25,8)	0,37 [0,19-0,68]	0,0007
no	110 (88,7)	184 (74,2)	1	
Number of prenatal visits (PNV)				
1	11 (10,6)	1 (0,4)	1	0
2-3	37 (35,6)	78 (32,3)	0,04 [0,005-0,4]	
≥4	56 (53,9)	162 (67,2)	0,03 [0,003-0,2]	
Mode of delivery				
vaginal delivery	68 (57,1)	90 (36,7)	2,4 [1,5-3,7]	0,0002
cesarean section	51 (42,9)	155 (63,3)	1	
Preeclampsia-eclampsia				
yes	19 (15,3)	31 (12,5)	1,3 [0,7-2,3]	0,8243
no	105 (84,7)	217 (87,5)	1	
Retroplacental hematoma				
yes	16 (12,9)	7 (2,8)	5,1 [2-12,8]	0
no	108 (87,1)	241 (97,2)	1	
Ectopic pregnancy				
yes	1 (0,8)	1 (0,4)	2 [0,1-32,4]	0,6264
no	123 (99,2)	247 (99,6)	1	
Placenta previa				
yes	2 (1,6)	3 (1,2)	1,3 [0,2-8,1]	0,7537
no	122 (98,4)	245 (98,8)	1	
Severe malaria				
yes	6 (4,8)	2 (1,2)	4,2 [1-16,9]	0,0388
no	118 (95,2)	245 (98,8)	1	

COR = Crude Odds Ratio

The variables independently associated with maternal death in the multivariate analysis were RPH (OR = 4.6; 95% CI [1.6–12.7]), ambulance transport (OR = 3.8; 95% CI [1.9–7.9]), and vaginal delivery (OR = 2.6; 95% CI [1.5–4.5]) (Table V).

**Table V:** Multivariate analysis of factors associated with maternal mortality at Tengandogo University Hospital, 2017–2022

Variables	Case n (%)	Control n (%)	COR [95%CI]	p	AOR [95%CI]	p
Mode of transport						
ambulance	106 (89,1)	136 (69)	3,7[1,9-7]	0	3,8 [1,9-7,9]	0, 000
personal vehicle	13 (10,9)	61 (31)	1		1	
Mode of delivery						
vaginal delivery	68 (57,1)	90 (36,7)	2,4[1,5-3,7]	0,0002	2,6 [1,5-4,5]	0
cesarean section	51 (42,9)	155 (63,3)	1		1	
Retroplacental hematoma						
yes	16 (12,9)	7 (2,8)	5,1 [2-12,8]	0	4,6 [1,6-12,7]	0,004
no	108 (87,1)	241 (97,2)	1		1	

COR = Crude Odds Ratio

AOR = Adjusted Odds Ratio

## Discussion

The primary objective of this study was to identify factors associated with maternal deaths at CHUT, a tertiary-level referral facility in the national healthcare system. It should be noted that this center admits patients evacuated or referred from first- and second-level healthcare facilities, including health and social promotion centers, medical centers with surgical units, regional hospitals, and private clinics, for specialized management of severe obstetric complications.

Data collection, conducted as part of a retrospective study, faced several methodological limitations. We sought to minimize data collection biases by using easily identifiable categorical variables and coding them (0 or 1) whenever possible. We excluded incomplete or missing medical records. Furthermore, for certain variables (mode of transport, occupation), data were missing. Additionally, the single-center nature of the study may have limited the generalizability of the results, although the CHUT receives patients from across the entire country. Since clinical practices, available resources, and hospitalization conditions may vary from one facility to another, these factors must be taken into account when interpreting the results.

Despite these limitations, we were able to calculate the indoor maternal mortality rate based on maternal deaths recorded from 2017 to 2022 at the CHUT and identify the factors associated with maternal deaths. The observed rate is high at this center, where the most severe obstetric complications are referred. This situation could also be due to the facility’s inadequate technical infrastructure (only one functional operating room, which contributes to longer wait times for the management of certain emergencies). Staff shortages necessitate sharing the on-call team with surgical staff. Added to this are, on the one hand, delays in consultation—or referral—for patients with complications and, on the other hand, an insufficient number of prenatal consultations [17].

Bouvier *et al.* and Say *et al.* have also described RPH as a risk factor [2,12]. Massive hemorrhage is a medical emergency requiring urgent care, the availability of labile blood products, and often, resuscitation measures. However, this is generally not the case in Burkina Faso, where qualified personnel and resuscitation equipment are insufficient. Since the study did not include response times for managing RPH, it is likely that these emergencies were treated beyond the optimal timeframe, as is often observed in our setting. Delays in the medical response, often associated with organizational and logistical constraints, may explain why RPH is significantly associated with maternal mortality.

Our study found an association between vaginal delivery and maternal death. Vaginal delivery itself is not risky, as most vaginal deliveries proceed without complications. According to the 2022 annual activity report from the CHUT, out of 2,758 deliveries, 1,195 were vaginal (43.32%), and 1,563 were C-sections. There were 59 maternal deaths across all delivery methods, representing 2% of deaths regardless of mode of delivery.

However, adverse outcomes for mothers and children can occur when obstetric complications are not detected in time during prenatal care or at delivery and when appropriate obstetric decisions are not made.

It is also important to consider whether the necessary products are available to handle certain emergencies. In our study, 15% of deceased patients had been admitted for preeclampsia or eclampsia. Maternal mortality audits at the CHUT had highlighted the unavailability of alpha-methyldopa. This forced family members to travel miles to purchase the medication, since the hospital is not located in the city. This may have increased the delay in care. Furthermore, 95.2% of patients were initially treated at a lower-level health facility before being referred due to a lack of technical infrastructure (e.g., no operating room, or an operating room that was out of service) and/or qualified personnel (e.g., lack of or insufficient number of gynecologists or intensivists, etc.). Unfortunately, our study did not assess the time between these patients’ admission to lower-level health facilities and the decision to refer them, even though this delay can influence the risk of death.

It is also important to mention the waiting time for ambulances, which are often insufficient in number. The association of ambulance transport with death is explained by a bias: ambulances transport the most severe cases. Several audits have shown that ambulances are insufficient, often out of service, and not staffed by medical personnel in most health facilities. Sometimes, several women are transported in the same ambulance. Strengthening the technical infrastructure of first- and second-level healthcare facilities and increasing the number of medicalized ambulances are essential for improving the management of obstetric emergencies and reducing maternal mortality.

Our results differ from those of Raoul *et al.*, who found that factors associated with maternal deaths included place of residence, a delay of five days or more between symptom onset and hospital admission, non-medicalized transportation, and “the third delay” [10]. Mahbouli *et al.* identified primiparity, multiparity, adverse socioeconomic conditions, and an insufficient number of prenatal visits as the main factors associated with maternal mortality [8]. Yego *et al.* described other factors, such as eclampsia, lack of prenatal care, and the presence of comorbidities, in an analytical study conducted in Kenya involving 450 women [16]. To reduce maternal mortality in Burkina Faso, the following strategies can be adopted: involving community health workers in recognizing danger signs, medicalizing obstetric referrals, and reducing the “third delay” (the decision to provide care) in referral facilities. This requires improving conditions for performing cesarean sections within a reasonable timeframe in cases of high-risk deliveries. The benefits of cesarean sections have already been highlighted by several authors [7,13,17].

## Conclusion

The indoor maternal mortality rate is high at the CHUT, a tertiary referral center. RPH, poor ambulance transport conditions, and complications related to vaginal delivery are factors that are independently associated with maternal deaths. To enable the obstetrics and gynecology department to operate autonomously, the CHUT must strengthen it in terms of human resources, both in quantity and quality.

Furthermore, having emergency supplies available is essential. Opening a second operating room would reduce waiting times for obstetric interventions. Healthcare providers at various first- and second-level healthcare centers (e.g., nurses, midwives, and gynecologists) must continuously strengthen their clinical skills and tools for the early diagnosis and proper management of obstetric complications. This will reduce the need for patient evacuations, which generally occur under inappropriate conditions. The same applies to managing peripartum or postpartum hemorrhage, which requires an improved supply of labile blood products. Finally, increasing the number of correctly equipped medical ambulances is strongly recommended.

## Acknowledgments

We thank the Director General of Tengandogo University Hospital for granting permission to collect data.

## Funding

This study received no funding.

## Authors’ contributions

Le Dr Vincent Kaboré a collecté les données dans le cadre de sa thèse d’exercice qui a été dirigée par le Dr Wedminère Noélie Zoungrana-Yameogo et le Pr Ali Ouedraogo. Les analyses et la rédaction de la version initiale ont été effectuées par la Dr Wedminère Noélie Zoungrana-Yameogo. La première version a été partagée avec Serge Alain Tougma, Abdoulaye Hama Diallo, Aristide Djiguimde, Dieudonné Hien, Anthony Some, Abdoulaye So, Fidèle Kafando, Mandeta Yankine, Aida Combary, Dominique Hélène Laurel Yabre et le Pr Ouedraogo Ali pour lecture.

## Conflicts of interest

The authors declare no conflicts of interest.
